# Stress Behaviour of an Immature Maxillary Central Incisor: A 3D Finite Element Analysis

**DOI:** 10.3390/ma18102305

**Published:** 2025-05-15

**Authors:** Petra Bučević Sojčić, Jasna Leder Horina, Nina Bočkaj, Tea Borojević Renić, Dubravka Turjanski, Kristina Goršeta, Tanja Jurčević Lulić, Hrvoje Jurić

**Affiliations:** 1 Department of Paediatric and Preventive Dentistry, School of Dental Medicine, University of Zagreb, Gundulićeva 5, 10 000 Zagreb, Croatia; gorseta@sfzg.unizg.hr (K.G.); juric@sfzg.unizg.hr (H.J.); 2 Independent Chairs, Chair of General Program Contents, Faculty of Transport and Traffic Sciences, University of Zagreb, Vukelićeva 4, 10 000 Zagreb, Croatia; jleder@fpz.unizg.hr; 3 Independent Researcher, 10 000 Zagreb, Croatia; ninabockaj@gmail.com; 4 Department of Prosthodontics, Dental Polyclinic Zagreb, Perkovčeva 3, 10 000 Zagreb, Croatia; tea5bor@yahoo.com; 5 Department of Pediatric and Preventive Dentistry, Dental Polyclinic Zagreb, Perkovčeva 3, 10 000 Zagreb, Croatia; dubravkadjuric81@gmail.com; 6 University Dental Clinic, University Hospital Centre Zagreb, Kišpatićeva 12, 10 000 Zagreb, Croatia; 7 Department of Applied Mechanics, Faculty of Mechanical Engineering and Naval Architecture, University of Zagreb, Ivana Lučića 5, 10 000 Zagreb, Croatia; tanja.jurcevic@fsb.unizg.hr

**Keywords:** maxillary central incisor, finite element analysis, post-endodontic treatment, root dentin

## Abstract

Background and Objective: Immature maxillary incisors (IMIs) are especially susceptible to failure due to their thin dentinal walls and compromised structural integrity following endodontic treatment. This study aims to evaluate the stress distribution within the root dentin after various post-endodontic treatments. Materials and methods: A personalized finite element analysis model of IMI was created using cone beam computed tomography (CBCT) data. Based on data from the literature, five stages of root development were reconstructed: half root development (S1), three-quarter development (S2), more than three-quarter development (S3), fully developed root with open apex (S4), and fully developed root with closed apex (S5). Six experimental groups were analyzed: GC Fiber Post (PS1); RelyX Post (PS2); metal post Unimetric 1.0 (PS3); everStick Post (PS4); positive control group with only the gutta-percha filling (PC), and intact maxillary incisor as negative control group (NC). The resulting equivalent stresses were evaluated using the Hencky–von Mises (HMH) strength theory. Results: The mean HMH stress within the root dentin was statistically significantly higher at the cervical level in all stages, except in stage S1 and models PS2 and PS3 in stage S2, where it was significantly higher at the apical level (*p* < 0.001 for all models, except stage S3 [PC model *p* < 0.005; NC model *p* < 0.008]). The PS4 model showed the lowest stress values at the cervical level in stages S1, S2, and S3 (55.19 MPa, 58.78 MPa, 58.84 MPa) and the PS1 model in stages S4 and S5 (57.48 MPa, 58.81 MPa). At the apical level, model PS3 showed the lowest stress values in stage S1 (69.60 MPa), model PS1 in stages S2, S3, and S5 (35.99 MPa, 44.30 MPa, 12.51 MPa) and model PC in stage S4 (17.85 MPa). Conclusions: The results showed that the greatest stress in an immature maxillary central incisor occurred at the cervical level, except during the early stage of root development. Post placement did not reduce root dentin stress.

## 1. Introduction

The management of non-vital immature maxillary incisors poses a major clinical challenge due to their underdeveloped root structures and compromised mechanical properties [1] requiring careful selection of treatment modalities to optimize the long-term outcomes [2]. The amount of remaining coronal structure and the residual thickness of the root dentin are critical factors in determining the appropriate type of final restoration [3,4,5,6]. In cases of severe coronal damage, the use of a post is often recommended to ensure adequate retention for the core and enhance the overall stability of the coronal restoration [3]. 

The development of esthetic, non-metallic post systems has influenced a shift from mechanical to adhesive post concepts [7]. Fiber-reinforced composite (FRC) posts are widely used in contemporary clinical practice due to their biomechanical compatibility with dentin [8]. Their modulus of elasticity is very close to that of natural dentin, promoting more favorable stress distribution and reducing the incidence of catastrophic failures such as post debonding and root fractures that extend beyond the alveolar bone [9]. Prefabricated glass fiber-reinforced composite posts are most commonly used due to their excellent esthetic properties and the formation of a monobloc with the tooth structure [10,11]. To minimize the need for extensive dentin removal in roots with larger canal diameters, individually shaped glass fiber-reinforced composite posts have been introduced. This approach reduces stress concentrations in the apical region while improving core support and overall structural integrity [12,13]. The direct application of composite restorations following post-endodontic treatment in the anterior region is increasingly favored as it preserves more healthy tooth structure and reduces the risk of root fracture [14], especially in pediatric patients where prosthetic rehabilitation remains controversial [15].

Clinical studies have shown that more than 50% of young permanent teeth with incomplete root development and necrotic pulp are lost in the first 10 years following endodontic treatment [16,17,18], which can also have a profoundly negative impact on a child’s psychosocial development [19]. Finite element analysis is widely recognized as a valuable tool for complex biomechanical investigations [20,21], which are crucial for understanding treatment failures and evaluating the performance of restorative materials [22,23]. Most finite element method (FEM) studies on maxillary incisors have reported that stress concentrations are usually located in the cervical region of the root [23,24,25,26,27,28,29,30,31]. In studies by Okamoto et al. [28] and Jain et al. [31], the highest stress values were recorded on the inner cervical side in models with FRC posts, while Nokar et al. [27] reported the highest stress concentrations between the middle and cervical third, however in mature incisors. Madfa et al. [32] reported that the cervical and apical region is where failure begins due to the high stresses between the post and the surrounding materials. Root immaturity has usually been defined in general terms (e.g., open apex, short root, weakened dentin wall) or related to a specific stage of development, usually the third stage of the Cvek classification [16]. However, no study has yet investigated the distribution of stress across all stages of root development.

The aim of this study was to assess stress distribution in immature maxillary incisor roots treated with various post systems and in relation to the developmental stage of the root using the finite element method.

The research hypotheses were:

1. The highest stress occurs in the cervical part of the root of an endodontically treated tooth with intracanal retention.

2. The intracanal retention and its stability depend on the thickness of the remaining root dentin.

## 2. Materials and Methods

### 2.1. Study Design

This study is an experimental in silico (computer-aided) investigation designed to evaluate the biomechanical performance of various post-endodontic restorative materials using the finite element method. Stress distribution within the root dentin was analyzed across several stages of development applying a comparative approach to evaluate six experimental groups. This study was conducted according to the guidelines of the Declaration of Helsinki, and approved by the Ethics Committee of the School of Dentistry, University of Zagreb, Croatia (approval number: 05-PA-27-5/2018). Written informed consent was obtained from the parents or legal guardians for the use of existing cone beam computed tomography (CBCT) images acquired primarily for diagnostic purposes related to orthodontic treatment prior to the start of this study. The consent form included detailed information about the objectives of this study, the methodology, and the significance of the expected results.

### 2.2. Model Generation

The geometry of an immature maxillary incisor (IMI) was isolated from a CBCT image of a school-aged child with an intact maxillary central incisor exhibiting completed root development. A three-dimensional tooth model was generated using the Mimics software package (version 16.0, Materialize, Leuven, Belgium). The finite element mesh was subsequently created using the Abaqus finite element analysis (FEA) software package (version 2020, Dassault Systemes, Simulia Corp., Johnston, RI, USA). Each part of the model was assigned its own finite element mesh, namely second-order tetrahedral finite elements consisting of ten nodes. The final output was a personalized 3D tooth model. The material properties, including the modulus of elasticity and Poisson’s ratio, are listed in Table 1. All model components were assumed to be linearly elastic and isotropic.

**Figure 1:**
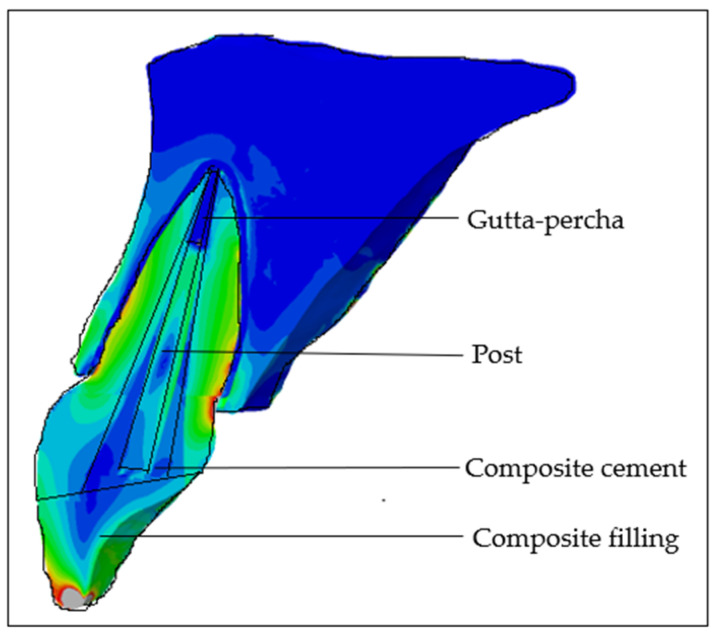
Schematic illustration of post-endodontic treatment.

**Figure 2:**
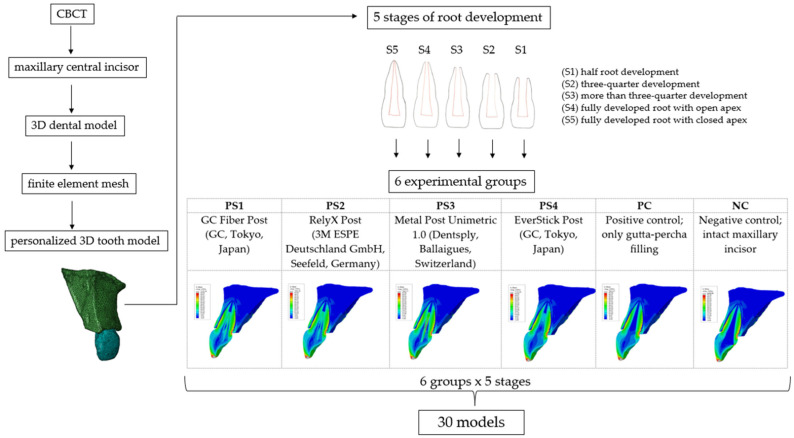
Schematic diagram of experimental groups.

Based on data from a previous pilot study [42], dentin thickness measurements from intraoral images of IMIs in children aged from 7 to 11 years were used to reconstruct the five stages of root development: half root development (S1), three-quarter development (S2), more than three-quarter development (S3), fully developed root with open apex (S4), and fully developed root with closed apex (S5). In the next step, a complicated oblique crown fracture involving the loss of more than half of the crown was simulated, where intracanal retention can be considered. The overall therapeutic approach simulated a post-endodontic treatment with intracanal retention and a minimally invasive composite restoration of the crown (Figure 1). The depth of post space preparation varied according to the stage of root development, with the main requirement being to leave 4 mm of gutta-percha in the apical portion of the root. The models were divided into experimental groups according to the type of post system used: GC Fiber Post (GC, Tokyo, Japan) (PS1); RelyX Post (3M ESPE Deutschland GmbH, Seefeld, Germany) (PS2); Metal Post Unimetric 1.0 (Dentsply, Ballaigues, Switzerland) (PS3); everStick Post (GC, Tokyo, Japan) (PS4); positive control group with only the gutta-percha filling (PC), and intact maxillary incisor as negative control group (NC) (Figure 2). 

The reconstruction involved modifying the mechanical properties within the root canal, which now included the properties of the gutta-percha, the post material, the cement, and the fractured crown segment restored with composite resin. The post was precisely positioned in the center of the root canal, maintaining a 4 mm distance between the apical foramen and the pulp chamber roof. The geometric specifications of each post were based on the manufacturer’s official documentation [43,44,45]. The remaining space surrounding the post and within the root canal was assigned the material properties of composite cement. In the case of the EverStick post, which is a customized post, it occupied almost the entire volume of the root canal, leaving 0.1 mm thick layer of composite cement surrounding the post. The simulation of the endodontic treatment included a gutta-percha filling up to 1 mm below the cervical margin, a 2 mm thick base layer of glass ionomer cement, and a composite restoration for the remaining portion of the crown filling. A total of 30 models were created.

### 2.3. Loading and Boundary Conditions

**Figure 3:**
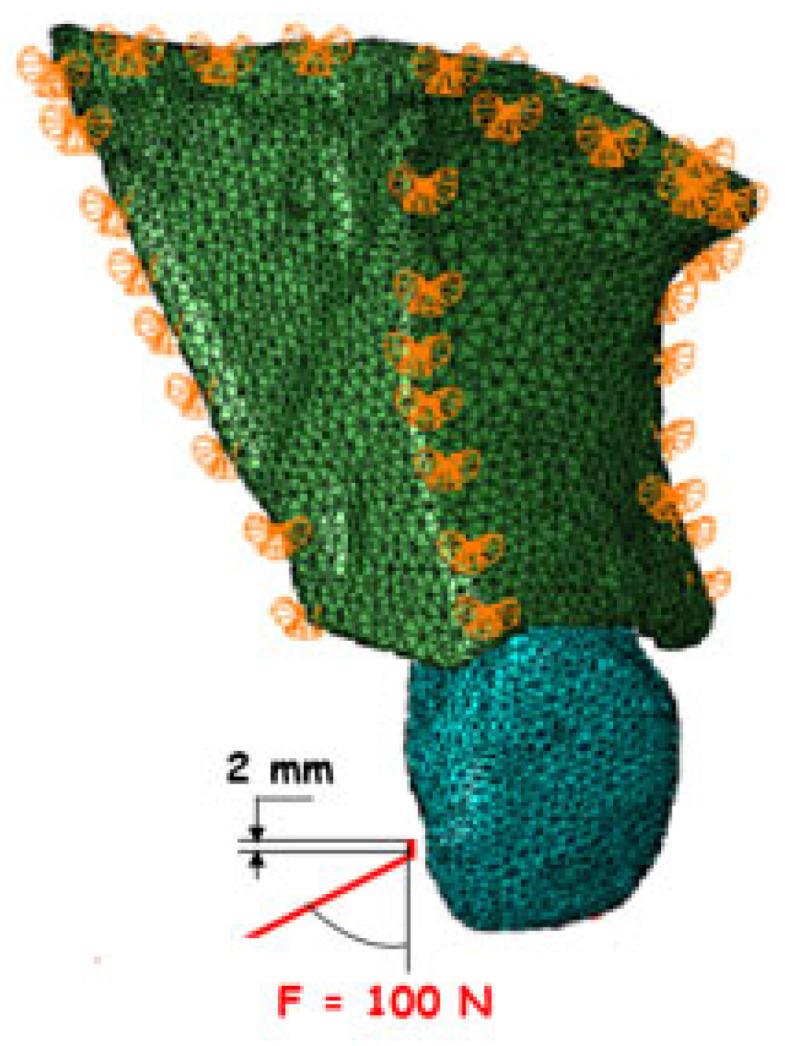
Loading and boundary conditions.

All directional movements were constrained to accurately simulate the anatomical connection with the rest of the maxilla. A static load of 100 N [46,47,48] was applied as a proportional continuous load to a small palatal surface of the simulated tooth at a 45-degree angle (Figure 3). The resulting equivalent stresses were evaluated using the Hencky–von Mises (HMH) stress theory.

### 2.4. Statistical Analysis

In all models, the highest stress concentrations were observed at the cervical and apical levels. Therefore, the 80 highest stress values from both levels were extracted for statistical analysis [49]. The middle level (between the apical and cervical levels) was not included as it did not show statistical significance in any of the groups. The normality of the data distribution within the individual groups of n = 80 was tested using the Shapiro–Wilk test and normal Q–Q plots. The analysis was performed using a three-way analysis of variance (ANOVA) with the factors “material”, “stage of development” and “root level (apical/cervical)”. For multiple comparisons, a one-way ANOVA with Tukey adjustment was used. The significance level was set at 0.05. Statistical analysis was performed with the SPSS software package, version 25.0 (IBM, Armonk, NY, USA).

## 3. Results

### Descriptive Statistics

The mean HMH stress within the root dentin was statistically significantly higher at the cervical level in all stages, except in stage S1 and in models PS2 and PS3 in stage S2, where it was significantly higher at the apical level (*p* < 0.001 for all models, except stage S3 [PC model *p* < 0.005; NC model *p* < 0.008]) (Table 2). The PS4 model showed the lowest stress values at the cervical level in stages S1 (55.19 ± 1.70 MPa), S2 (58.78 ± 0.93 MPa), and S3 (58.84 ± 1.04 MPa), while the PS1 model showed the lowest values in stages S4 (57.48 ± −1.03 MPa) and S5 (58.81 ± 1.08 MPa). At the apical level, the lowest stress values were observed in the PS3 model at stage S1 (69.60 ± 5.03 MPa), in the PS1 model at stages S2 (35.99 ± 2.63 MPa), S3 (44.30 ± 4.07 MPa), and S5 (12.51 ± 1.42 MPa) and in the PC model at stage S4 (17.85 ± 1.88 MPa). Stress within the root dentin showed a decreasing trend from stage S1 to stage S5 in most models at both levels. However, notable deviations were observed in the PS1 and NC models, where stage S2 at the apical level exhibited significantly lower stress than stage S3, in the PS3 model, where stage S1 at the apical level exhibited significantly lower stress than stage S2, and in the PS4 model, where stage S1 at the cervical level exhibited significantly lower stress than stage S2 and all subsequent stages. In general, no statistically significant differences were observed between the post systems at both levels for all stages, especially for the stages with the highest clinical relevance (S4 and S5) (Table 3).

**Figure 4:**
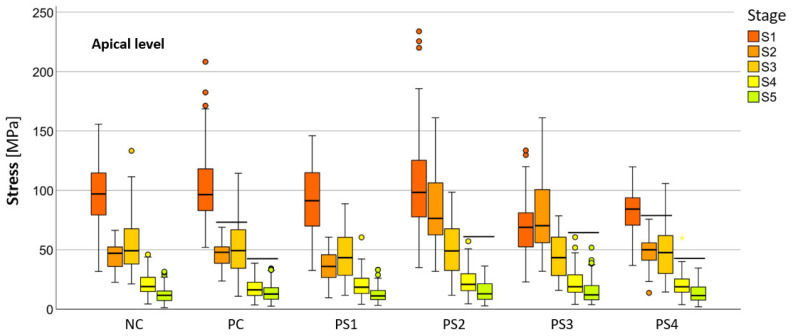
Distribution of HMH stress between models divided into five stages of root development at the apical level (SPSS software package, version 25.0, IBM, Armonk, NY, USA).

**Figure 5:**
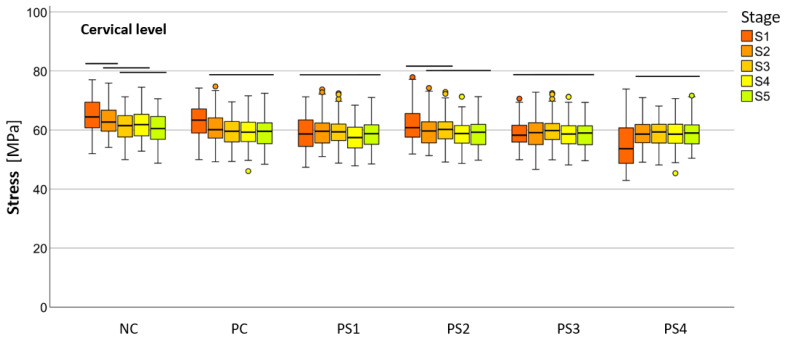
Distribution of HMH stress with a smaller range on the Y-axis (0-100 MPa) between models divided into the five stages of root development at the cervical level (SPSS software package, version 25.0, IBM, Armonk, NY, USA).

The numerical values of the equivalent stresses according to the HMH theory within the root dentin, categorized into five stages of root development at two levels for all materials, are shown in Figure 4 and Figure 5.

**Figure 6:**
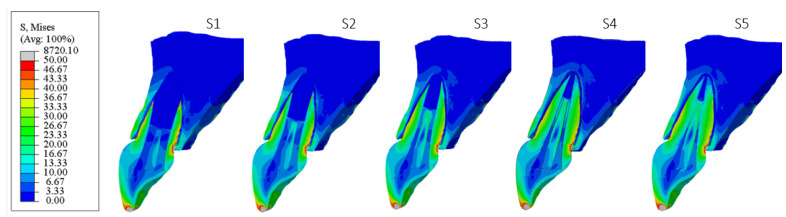
PS1 model according to the root development stage—sagittal section.

**Figure 7:**
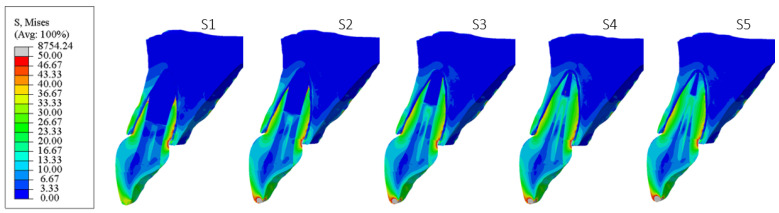
PS2 model according to the root development stage—sagittal section.

**Figure 8:**
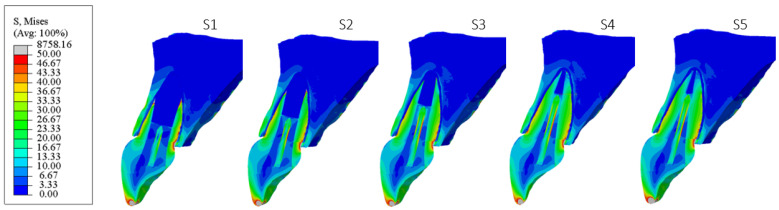
PS3 model according to the root development stage—sagittal section.

**Figure 9:**
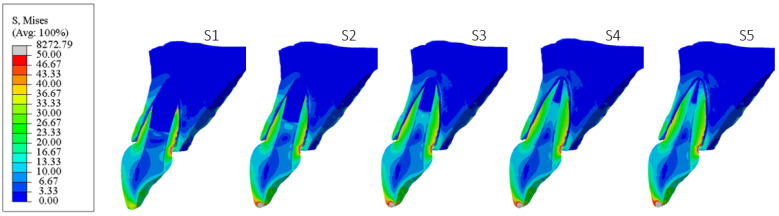
PS4 model according to the root development stage—sagittal section.

**Figure 10:**
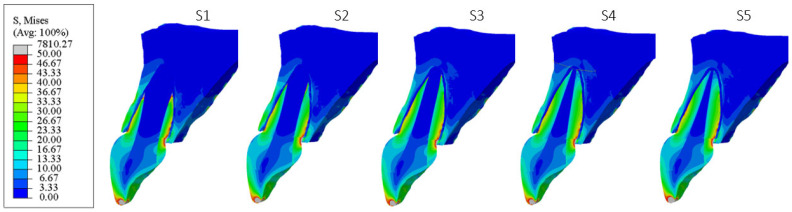
PC model according to the developmental stage of the root—sagittal section.

**Figure 11:**
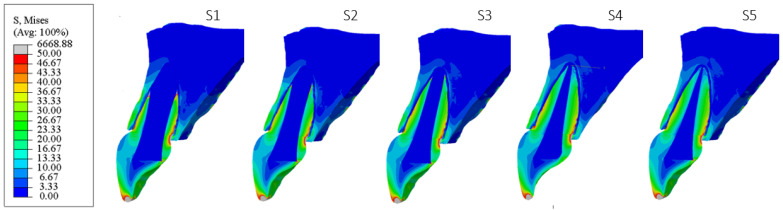
NC model according to the developmental stage of the root—sagittal section.

Figure 6, Figure 7, Figure 8, Figure 9, Figure 10 and Figure 11 show sagittal cross-sections of the individual finite element analysis models with respect to the different stages of root development.

## 4. Discussion

There are many FEM studies that have evaluated endodontically weakened or immature maxillary incisors, but given the different protocols and treatments, the results of this study could only be compared with a few.

HMH stress was highest at the cervical level in most models, which is consistent with findings in the literature [23,26,29,30,31]. Jain et al. [31] reported maximum stress concentration in the cervical third of the root when simulating two no-post systems with a decrease in stress intensity from the cervical to the apical third. Eram et al. [26] observed higher stresses in the cervical portion of the materials used for the apical stop simulating the third stage of Cvek on an extracted adult incisor before CBCT examination. Similarly, Gurbuz et al. [29] reported the highest stresses in the cervical region of the root dentin, but in primary central incisors when different composite materials were used to form a short post and core. Their maximum stress values (83.0–85.8 MPa) were higher than those observed in our study (59.44–63.00 MPa) at the same level. This discrepancy may be attributed to differences in anatomical properties, particularly the much higher crown-to-root ratio in primary incisors and the shorter length of the composite material used for the post function, as well as poorer mechanical properties compared to FRC posts. In the study by Kumar et al. [30], the highest stress areas in FRC posts were at the inner proximal wall at the cervical level (15.33–15.37 MPa) and at the inner apical side of the metal posts (14.92–15.01 MPa) in FEA models of IMI with completed root development, and therefore we could only compare their values with our S5 stage (58.81 MPa at the cervical level and 12.51 MPa at the apical level for the PS1 model and 58.66 MPa at the cervical level and 15.17 MPa at the apical level for the PS3 model). It is noteworthy that their values at the apical level are the same as ours, while those at the cervical level are 3 to 4 times higher. This could be due to differences in the approach to modeling the immature maxillary incisor. In their study, the FEA model was created by scanning the extracted maxillary incisor of an adult, whereas in our study, the FEA model was developed from CBCT data of an 11-year-old child. This highlights the methodological challenges of modeling the immature maxillary central incisor and the ethical considerations of evaluating therapeutic procedures in an in vivo model.

Manaktala et al. [50] investigated the stress distribution in endodontically treated maxillary incisors with external cervical resorptions restored with mineral trioxide aggregate (MTA) and Biodentine. The maximum stresses were found in the cervical region of the dentin and were also similar between intact and restored incisors, as observed in our study. This suggests that the cervical region remains a critical point for stress concentration, regardless of whether the tooth is intact or restored, highlighting the need for careful evaluation of the long-term durability of restorative materials in this area. Hassouneh et al. [23] assessed different therapeutic approaches in non-vital IMI at the third stage of Cvek and also reported the highest stress concentration in the cervical area. Their values for stress concentration in the glass fiber post (GFP) model (64.1 MPa) were comparable to those in our PS1 model in S3 stage (59.3 MPa). In addition, their intact model exhibited the highest stress values (90.3 MPa) similar to our NC model in S3 stage (61.4 MPa). However, in contrast to our findings, they reported that the use of GFP improved biomechanical performance. The larger deviations in stress values could be attributed to the higher load (240 N) used to simulate occlusal forces in their study. 

Based on the above-mentioned studies on maxillary central incisors following post-endodontic treatment, it can be concluded that the highest stress concentrations typically occur in the cervical region, regardless of different modeling techniques or the stage of root development. However, there is lack of data in the literature regarding the very early stages of root development. In our study, the stress concentrations in these early stages were highest in the apical region, likely due to more pronounced change in the thickness of the root dentin. We believe that future studies will further validate the observed pattern of stress distribution. In addition, differences in geometric modeling between studies resulted in stress values that were not always directly comparable, emphasizing the need for a standardized protocol.

Comparison of stress concentrations in root dentin between developmental stages within each group in our study showed a decrease from S1 to S5, as expected given the progressive thickening of the dentin wall at each subsequent stage. The deviations observed in stages S2 and S3 can be attributed to differences in the modeling of these stages based on our pilot study [42]. In particular, a value corresponding to two-thirds of root development was used for stage S2, whereas a range between S2 and S4 (more than two-thirds of development) was applied for stage S3, which may have contributed to greater variability in the modeling.

A comparison between the model with intact tooth and the model with gutta-percha filling revealed no significant differences in the stress distribution within the root dentin. This finding supports the assumption that endodontic treatment alone has no significant influence on the stability of the tooth, consistent with previous reports in the literature [51,52]. Furthermore, the comparison between the gutta-percha model and the models with post retention in stages S4 and S5, where such restorations are most commonly used clinically, showed no significant differences in the stress concentrations at the either cervical or apical level of the root dentin. These results support a minimally invasive post-endodontic approach and emphasize the need to carefully evaluate the clinical benefits of intracanal retention. Alshabib et al. [7] observed an increasing trend towards the use of endocrown restorations and extended onlays that do not rely on a post-core system, instead utilizing a box-shaped preparation to enhance retention of the crowns or onlays. The long-standing debate among clinicians about the necessity of posts, often formulated as “To post or not to post?” [14,53,54,55], remains unresolved, especially in young permanent teeth, where clinical research is inherently challenging. In this context, finite element analysis can be a valuable tool to support the selection of appropriate therapeutic strategies [56,57].

All root development models restored with the GC Fiber Post system showed lower stress concentrations within the root dentin at the apical level compared to those restored with RelyX Post, suggesting that the geometry of the GC Fiber Post, which has a cylindrical base coronally and a tapered end apically, more closely follows the natural anatomy of the tooth [12,58,59]. Nevertheless, post placement did not reduce root dentin stress. Anthrayose et al. [60], using a similar protocol that simulated only Cvek stage 3, also reported no significant differences between the immature intact model (106.18 MPa) and the immature models subjected to apexification or revascularization (114.23 MPa), findings that are consistent with the results of our models.

No major deviations were observed in metal posts compared to esthetic post systems, which is in contrast to reports in the literature [61,62,63]. Interestingly, some FEM studies have shown that higher stresses occur in glass fiber-reinforced composite post than in a metal post [27,30]. Furthermore, it should be considered that cyclic mechanical loading would provide a more appropriate approach for evaluating the long-term performance of metal posts, as it would likely result in significantly higher stress concentrations. This consideration is particularly relevant given the weaker bond strength between metal posts and composite cement, which increases the risk of irreparable root fractures extending below the bone level, caused by the high modulus of elasticity [53,64]. Notably, in all models with metal posts, stress accumulation was consistently observed in the middle third of the post on the oral side, a result that was not seen in any of the groups with esthetic post systems.

The comparison between the customized (PS4 model) and the prefabricated aesthetic post system (PS1 model) showed insignificant differences in the stress concentration within the root dentin, except in the S1 stage at the apical level, which showed significantly lower values in the PS4 model. The Everstick Post does not require additional removal of tooth structure and, unlike other aesthetic posts, features individualized geometry that adapts to the shape of the canal. Furthermore, as a relatively new system, the mechanical properties required for an accurate FEM analysis have not yet been thoroughly investigated in the literature. Gomes et al. [65] pointed out the possible unreliability of FEM results if inappropriate values for the material properties are used. We believe that further research is needed to obtain more accurate values for mechanical properties of the Everstick post system and to update the data for other materials as well, recognizing that this process requires complex and time-consuming laboratory techniques.

In summary, both hypotheses were confirmed, except at the earliest stage of root development, where the higher HMH stress was at the apical level.

This study has some limitations. A single CBCT was used for this study to obtain a personalized model of a tooth with completed root development, while the stages of root development and therapeutic procedures were modeled using the literature data [66] and dentin wall measurements on intraoral radiographs [42]. Better model properties would be achieved with a CBCT that follows the growth and development of teeth from the same patient, which would be highly ethically questionable in children. Since all model components were defined as linearly elastic and isotropic, the numerical simulation did not fully replicate the real in vivo conditions of structures and dental materials. A perfect adhesive bond between the materials was assumed. The mechanical properties of the structures and materials used were taken from a large number of published studies. Additional tests are required to obtain more accurate values. Finally, there were no data in the literature with which our results could be fully compared, as no single study used the same protocol for modeling root immaturity, nor were all stages of root development of the maxillary central incisor evaluated. Therefore, our results represent a special contribution to this field of science.

Future research in this area should focus on the clinical validation of the proposed modeling protocol, alongside the development of more advanced and physiologically accurate material models. In addition, ongoing efforts to reach a standardized consensus on modeling protocols are essential. Furthermore, a more in-depth investigation into the mechanical properties of the materials is required.

It is also important to emphasize that CBCT scans should not be performed solely for research purposes unless they are part of a clinically justified and previously indicated diagnostic or therapeutic procedure. In summary, strict adherence to ethical and legal standards is essential when using CBCT imaging in dental studies involving pediatric patients.

## 5. Conclusions

Despite the limitations of this study, it can be concluded that the greatest stress in an immature maxillary central incisor occurred at the cervical level, except during the early stage of root development. Post placement did not reduce root dentin stress.

The Everstick Post was found to be the most suitable material in the early stages of root development, while the GC Fiber Post was found in the later stages, at the cervical level. The post design with a cylindrical coronal base and a tapered apical end demonstrated better performance when selecting an aesthetic prefabricated root canal post compared to a fully conical post design. In addition, the metal post did not result in a significantly higher development of HMH stresses within the root dentin.

## Figures and Tables

**Table 1:** Mechanical properties.

Structure/Material	*E*, GPa	ν, -	References
Enamel	41.0	0.30	[33]
Dentin	18.6	0.31	[33]
Periodontal ligament (PDL)	0.00067	0.45	[34]
Pulp	0.00207	0.45	[33]
Cortical bone	13.4	0.26	[33]
Trabecular bone	0.345	0.36	[33]
Gutapercha	0.14	0.45	[35]
Glass ionomer cement	8.0	0.25	[36]
Composite filling	13.45	0.17	[37]
Composite cement	8.3	0.28	[38]
PS1 (GC Fiberpost)	40.0	0.25	[39]
PS2 (RelyX post 3M ESPE)	37.5	0.26	[40,41]
PS3 (Metal post Unimetric 1.0 Dentsply)	110.0	0.32	[38]
PS4 (EverStick Post GC)	15.0	0.18	[39]

**Table 2:** Mean value of Hencky–von Mises (HMH) stress [MPa] ± 95% confidence interval and standard deviation (SD) at each stage of root development at the apical and cervical levels.

APICAL LEVEL						
Model		S1	S2	S3	S4	S5
PS1	Mean	91.78 ± 6.17	35.99 ± 2.63	44.30 ± 4.07	20.12 ± 2.14	12.51 ± 1.42
	SD	27.75	11.82	18.33	9.64	6.42
PS2	Mean	105.59 ± 8.72	85.50 ± 6.84	51.11 ± 4.55	23.29 ± 2.47	15.19 ± 1.95
	SD	39.19	30.74	20.47	11.13	8.78
PS3	Mean	69.60 ± 5.03	80.84 ± 6.87	44.53 ± 4.08	21.73 ± 2.53	15.17 ± 2.33
	SD	22.61	30.87	18.37	11.40	10.50
PS4	Mean	82.83 ± 3.95	48.43 ± 2.73	47.95 ± 4.71	20.24 ± 2.14	14.08 ± 1.81
	SD	17.77	12.29	21.19	9.63	8.17
PC	Mean	103.44 ± 6.68	46.10 ± 2.32	52.49 ± 4.89	17.85 ± 1.88	14.40 ± 1.84
	SD	30.05	10.45	22.01	8.46	8.28
NC	Mean	97.95 ± 5.95	44.96 ± 2.39	54.28 ± 5.20	21.25 ± 1.96	12.67 ± 1.63
	SD	26.76	10.77	23.36	8.82	7.35
**CERVICAL LEVEL**						
Model		S1	S2	S3	S4	S5
PS1	Mean	59.06 ± 1.28	59.70 ± 1.11	59.35 ± 0.79	57.48 ± 1.03	58.81 ± 1.08
	SD	5.77	5.01	4.97	4.65	4.88
PS2	Mean	61.60 ± 1.25	60.12 ± 1.14	59.89 ± 1.14	58.79 ± 1.02	58.98 ± 1.09
	SD	5.63	5.16	5.14	4.59	4.91
PS3	Mean	58.77 ± 0.96	59.29 ± 1.13	59.64 ± 1.11	58.62 ± 1.08	58.66 ± 0.98
	SD	4.34	5.11	5.02	4.87	4.42
PS4	Mean	55.19 ± 1.70	58.78 ± 0.93	58.84 ± 1.04	58.74 ± 1.11	58.87 ± 1.02
	SD	7.67	4.19	4.68	5.03	4.58
PC	Mean	63.00 ± 1.24	60.51 ± 1.20	59.64 ± 1.08	59.44 ± 1.14	59.50 ± 1.14
	SD	5.58	5.42	4.85	5.12	5.14
NC	Mean	64.88 ± 1.33	63.12 ± 1.06	61.42 ± 1.13	62.07 ± 1.13	60.68 ± 1.10
	SD	6.01	4.77	5.12	5.11	4.96

**Table 3:** Results of univariate ANOVA comparing different types of materials between developmental stages at the apical and cervical level (Tukey HSD).

*p*-VALUE											
		APICAL LEVEL					CERVICAL LEVEL				
Model		S1	S2	S3	S4	S5	S1	S2	S3	S4	S5
PS1	PS2	0.025	0.000	0.299	0.330	0.325	0.075	0.995	0.983	0.543	1.000
	PS3	0.000	0.000	1.000	0.909	0.333	1.000	0.995	0.999	0.687	1.000
	PS4	0.337	0.001	0.874	1.000	0.842	0.001	0.848	0.986	0.586	1.000
	PC	0.094	0.019	0.125	0.697	0.706	0.000	0.906	0.999	0.120	0.94
	NC	0.735	0.055	0.029	0.979	1.000	0.000	0.000	0.091	0.000	0.141
PS2	PS1	0.025	0.000	0.299	0.330	0.325	0.075	0.995	0.983	0.543	1.000
	PS3	0.000	0.683	0.339	0.919	1.000	0.031	0.897	0.999	1.000	0.998
	PS4	0.000	0.000	0.929	0.372	0.959	0.000	0.524	0.759	1.000	1.000
	PC	0.997	0.000	0.998	0.007	0.991	0.666	0.996	1.000	0.960	0.983
	NC	0.520	0.000	0.927	0.783	0.397	0.007	0.002	0.376	0.000	0.224
PS3	PS1	0.000	0.000	1.000	0.909	0.333	1.000	0.995	0.999	0.687	1.000
	PS2	0.000	0.683	0.339	0.919	1.000	0.031	0.897	0.999	1.000	0.998
	PS4	0.036	0.000	0.903	0.931	0.962	0.002	0.987	0.912	1.000	1.000
	PC	0.000	0.000	0.148	0.133	0.992	0.000	0.628	1.000	0.898	0.879
	NC	0.000	0.000	0.036	1.000	0.406	0.000	0.000	0.207	0.000	0.087
PS4	PS1	0.337	0.001	0.874	1.000	0.842	0.001	0.848	0.986	0.586	1.000
	PS2	0.000	0.000	0.929	0.372	0.959	0.000	0.524	0.759	1.000	1.000
	PS3	0.036	0.000	0.903	0.931	0.962	0.002	0.987	0.912	1.000	1.000
	PC	0.000	0.977	0.736	0.651	1.000	0.000	0.234	0.911	0.946	0.962
	NC	0.010	0.884	0.383	0.987	0.894	0.000	0.000	0.014	0.000	0.169
PC	PS1	0.094	0.019	0.125	0.697	0.706	0.000	0.906	0.999	0.120	0.944
	PS2	0.997	0.000	0.998	0.007	0.991	0.666	0.996	1.000	0.960	0.983
	PS3	0.000	0.000	0.148	0.133	0.992	0.000	0.628	1.000	0.898	0.879
	PS4	0.000	0.977	0.736	0.651	1.000	0.000	0.234	0.911	0.946	0.962
	NC	0.820	0.999	0.994	0.254	0.779	0.338	0.012	0.208	0.009	0.636
NC	PS1	0.735	0.055	0.029	0.979	1.000	0.000	0.000	0.091	0.000	0.141
	PS2	0.520	0.000	0.927	0.783	0.397	0.007	0.002	0.376	0.000	0.224
	PS3	0.000	0.000	0.036	1.000	0.406	0.000	0.000	0.207	0.000	0.087
	PS4	0.010	0.884	0.383	0.987	0.894	0.000	0.000	0.014	0.000	0.169
	PC	0.820	0.999	0.994	0.254	0.779	0.338	0.012	0.208	0.009	0.636

## Data Availability

All the data used in this study are available on request from the corresponding author.

## Abbreviations

The following abbreviations are used in this manuscript:

ANOVA	Analysis of variance
CBCT	Cone beam computed tomography
FEA	Finite element analysis
FEM	Finite element method
FRC	Fiber-reinforced composite
GFP	Glass fiber post
HMN	Hencky–von Mises
IMI	Immature maxillary incisor
NC	Negative control group—intact maxillary incisor
PC	Positive control group with only the gutta-percha filling
PS1	Post system with GC Fiber Post
PS2	Post system with RelyX Post
PS3	Post system with metal post Unimetric
PS4	Post system with GC everStick Post
S1	Stage with half of the root development
S2	Stage with three quarters of root development
S3	Stage with more than three quarters of root development
S4	Stage with fully developed root with open apex
S5	Stage with fully developed root with closed apex
